# Naming: What Do We Know So Far? A Systematic Review

**DOI:** 10.1007/s40614-023-00374-1

**Published:** 2023-05-12

**Authors:** Maithri Sivaraman, Dermot Barnes-Holmes

**Affiliations:** 1 School of Psychology, Cardiff University, Cardiff, UK; 2 School of Psychology, Ulster University, Coleraine, UK

**Keywords:** naming, verbal behavior development, bidirectional naming, listener behavior, speaker behavior

## Abstract

Although the term *naming* is used colloquially in the English language, it refers to a specific instance of verbal behavior within behavior analysis. Since Horne and Lowe’s (Horne & Lowe, 1996) seminal account on naming, the concept continues to generate clinical and research interest to-date. We conducted a systematic search of the behavior analytic studies on naming to highlight the methods that were used to test naming, the terminology that have been adopted, the conceptual underpinnings, and the methods used to train naming if it was found to be absent. Forty-six studies met inclusion criteria and we conducted a descriptive analysis of these studies. We found that most studies either used the terms naming or bidirectional naming. We found wide variation in the methods used to test and train naming. Nearly one third of these studies attempted to offer evidence that naming facilitated some other type of behavior, and the remaining studies attempted to train naming in individuals when the behavior was found to be absent. Overall, our review highlighted that there exists a rich empirical dataset on testing and training naming within behavior analysis, and we discussed specific areas for future research.

Children learning the names of objects around them is deemed an important area of study both within and outside behavior analysis. In the natural environment, young children encounter novel objects during interactions with their caregivers. These interactions may involve caregivers presenting an object to the child along with its name. Although the usage of the term *naming* is commonplace, behavior analysts frequently use naming as a technical concept to refer to a specific instance of verbal behavior. In particular, when a child with naming is exposed to a novel object and its name (e.g., “Look, here’s a dolphin”), they orient towards the object upon hearing its name (i.e., emit listener behavior), and also state the object’s name (i.e., speaker behavior) at a later point in time. Young children between 2 and 4 years of age have been shown in behavior analytic studies to demonstrate naming in the technical sense described above (see, for example, Gilic & Greer, 2011; Lowe et al., 2002).

## General Background to Naming Research

Horne and Lowe (1996) were one of the earliest behavior-analytic researchers to focus on naming, and their theory appears to be primarily driven by an attempt to explain equivalence relations^1^ as defined by Sidman and colleagues (e.g., Sidman et al., 1982). The details of Horne and Lowe’s naming theory are complex, but in essence they noted that reinforcement of a listener response produced a speaker response and vice-versa without additional training. Their theory was an extension of Skinner’s (1957) work and described naming as the integration of the listener and speaker within an individual and how this comes about. Subsequent empirical studies were conducted that showed that naming facilitated equivalence class formation (Carr & Blackman, 2001) and categorization in general (e.g., Lowe et al., 2005), and this made naming an important aspect of language curriculums for children, particularly those with developmental delays (Miguel & Petursdottir, 2009). verbal behavior development theory (VBDT) added to Horne and Lowe’s account by emphasizing naming as being the source of learning language *incidentally* (Greer & Keohane, 2005; Greer & Longano, 2010), and focused specifically on *how* the fusion of the listener and speaker occurs within an individual. Horne and Lowe’s series of studies aimed at identifying whether naming facilitated categorization and equivalence, and VBDT studies were focused on the establishment of naming itself. This line of research has yielded studies targeting techniques to produce naming in children, and evaluations on the progression from what Greer and colleagues refer to as preverbal foundational cusps to complex verbal capabilities including naming, reading, and writing (Greer & Ross, 2008).

Some naming theorists have argued that naming constitutes genuine verbal behavior (Greer & Longano, 2010; Horne & Lowe, 1996). This argument is in line with relational frame theory (RFT), an overarching behavior-analytic account of human language and cognition (Barnes-Holmes et al., 2020; Hayes et al., 2001). According to RFT, the relational responding involved in naming itself could be considered a class of generalized operant behavior. As such, naming may be characterized as a contextually controlled derived bidirectional relation between an object and its name. That is, the sight of a dolphin and the spoken word “dolphin” may be related by contextual cues for the frame of coordination such as “This is a” or the caregiver pointing towards the object. It is important to note, however, that relational frame theorists and naming theorists agree on the centrality of naming as being fully or genuinely verbal (Greer & Longano, 2010; Hayes et al., 2001).

Furthermore, studies that approached naming from the VBDT perspective have demonstrated that children with naming learn faster and in new ways compared to others who do not meet criterion for naming. For example, Greer et al. (2011) showed that children’s rate of learning measured during regular instructional activities accelerated once naming was established. Hranchuk et al. (2019) observed that when children had acquired naming, instructional demonstrations were more efficient than standard teaching trials that involved the presentation of programmed consequences. That is, children with naming performed correctly on mathematics tasks (e.g., “put these numbers in the right order”) simply by observing an instructor demonstrating the correct response. Therefore, these researchers have argued that naming is a verbal developmental cusp.

## Recent Terminological Issues

Although behavior analysts’ interest in naming has remained strong over the years, the terminology used in naming research has undergone changes. Horne and Lowe (1996) originally described the behavior as naming. They acknowledged that the term was commonly used in the English language and by scientists of allied disciplines including psychology and linguistics, but hoped that this would foster productive communication across disciplines. Miguel (2016), however, noted the confusion caused by the commonsense use of the term and proposed adopting the phrase c*ommon bidirectional naming*. This was an attempt to differentiate the behavior from the colloquial use of the term naming and to alert readers to its technical definition within behavior analysis.

Hawkins et al. (2018) offered a subsequent classification of six different types of naming. They aimed to discriminate between the emergence of untaught listener/speaker behavior (i.e., one of the topographies was trained, and the other emerged without training) and the acquisition of new names without programmed consequences for either topography of responses. The former category involved three types of naming: listener unidirectional naming (i.e., speaker behavior was trained and listener emerged), speaker unidirectional naming (i.e., listener behavior was trained and speaker emerged), and bidirectional naming (speaker behavior was trained and listener emerged, and vice-versa when tested with a novel set each). Likewise, the acquisition of listener and speaker behavior without programmed consequences for either topography also involved three subtypes of naming. The authors defined listener incidental unidirectional naming (listener behavior emerged from simply pairing objects with their names; but not speaker behavior), speaker incidental unidirectional naming (speaker behavior emerged from simply pairing objects with their names; but not listener behavior), and incidental bidirectional naming (both listener and speaker behavior emerged from simply pairing objects with their names). Thus, the existing literature seems to propose several terms to describe naming and its subtypes, but the extent to which these terms will be employed in the wider literature, and their value in terms of technical precision, remains to be seen. For the purposes of the current review, we will employ the generic term “naming” to refer to what Miguel (2016) calls *common bidirectional naming* but distinguish between listener and speaker responses when discussing specific experimental procedures and results.

## A Key Conceptual Issue

In terms of one of the primary aims of the current article, it seems important to return to the early history of naming research. In particular, as noted above, the initial research agenda on naming seemed to emerge out of Sidman’s (Sidman, 1971; Sidman et al., 1982; Sidman et al., 1986) seminal account on stimulus equivalence. Although empirical studies demonstrated that humans with verbal abilities readily pass equivalence tests, nonhuman species failed to consistently show such behavior (e.g., Dugdale & Lowe, 2000). Further, theoretical arguments put forth at the time suggested that the existence of equivalence relations in an individual’s repertoire could account, in a functional sense, for symbolic reference or semantic meaning (Sidman et al., 1982). In contrast to Sidman’s position that equivalence provides the basis for symbolic reference, Horne and Lowe’s (1996) account of naming was proposed as an explanation for equivalence. In particular, they argued that success during equivalence tests was largely due to naming and other verbal behavior, thereby explaining why nonhumans could not pass these tests.

Thus, much of the original research agenda born out of the naming account was largely focused on demonstrating that naming (i.e., listener and speaker behavior) was central to the establishment of equivalence and categorization (e.g., Lowe et al., 2002; Lowe et al., 2005). These original studies tested naming by training one of the response topographies. That is, researchers trained listener behavior and tested participants for the emergence of speaker behavior, or vice-versa. These studies showed that when children demonstrated naming they passed tests of categorization and/or stimulus equivalence. This experimental strategy (i.e., training one of the topographies, and testing for the emergence of the other) has been extended to children with autism and developmental delays (e.g., Miguel & Kobari-Wright, 2013) and to educational stimuli (Miguel & Petursdottir, 2009)

Research on naming has extended beyond the early focus on its role in stimulus equivalence and categorization. For example, children’s *incidental* learning of novel names simply by hearing caregivers label an object with its name was not targeted specifically in the early studies. However, a specific research agenda focused on the incidental learning of names seems to have emerged from Hart and Risley’s (1995, 1999) series of studies and VBDT (Greer & Keohane, 2005). This line of research typically involves a *naming experience*—an observational experience in which children hear a name in the visual presence of an object (e.g., Greer et al., 2005). Children are then tested on their listener and speaker naming responses towards this object without any programmed consequences (Greer et al., 2005; Pérez-González et al., 2014).

## Methods to Test and Train Naming

In our initial search of the naming literature, we observed key differences in the methods used to test naming. For instance, some studies administered a naming test with one object at a time (e.g., Luciano et al., 2007) whereas others used multiple objects per test administration (Pérez-González et al., 2014). Next, most previous research on naming has involved presenting the object and its name simultaneously. A recent study argued that when objects and their names are presented simultaneously, there may be no need to invoke a derived relation to explain emergent responding (see Sivaraman et al., 2021, for a detailed analysis). That is, the object and name are presented contemporaneously and any emergent relation could be seen as being directly trained because the child *sees object–hears name* and *hears name–sees object* during the presentation. In contrast, if naming responses emerge when objects and their names are presented nonsimultaneously (i.e., an object is presented first, hidden upon visual contact from the child, and then its name is stated), then at the very least a derived bidirectional relation may be involved. Furthermore, children seem to demonstrate naming even when objects and their names are presented with long delays. For example, during a car ride, a caregiver may say “did you see that? That was a fort” after the fort has disappeared from view. It seems important to study how naming is established under such conditions.

To the best of our knowledge, there have been no previous attempts to characterize the type of stimulus presentation method (i.e., simultaneous or nonsimultaneous, type and number of stimuli) used in the naming literature. Highlighting these characteristics seems important for both theoretical and practical reasons, to improve precision in the description of the variables manipulated to study naming, and the methods used during the testing and training of naming for better replicability of previously reported findings.

In addition, the applied behavior analytic literature presents a series of studies investigating methods to train naming when children do not demonstrate the behavior. For example, Greer et al. (2005) tested a procedure to train bidirectional naming in three preschool children. The procedure was termed multiple exemplar instruction and involved training children in multiple response topographies such as identity matching, pointing to, and labelling items across stimulus sets until they demonstrated correct responding across a novel set without training. Other studies have evaluated how establishing specific stimuli as reinforcers using conditioning procedures affects bidirectional naming (e.g., Longano & Greer, 2014; and Olaff & Holth, 2020, tested the impact of conditioning social stimuli as reinforcers). There have also been studies describing instructional procedures to establish bidirectional naming and how this induces categorization skills in children (e.g., Lee et al., 2015). Overall, although there have been multiple studies evaluating procedures to train naming, there seem to have been no attempts made to summarize the types or the efficacy of the aforementioned procedures.

## The Current Review

A rich vein of research has been generated, therefore, on naming within behavior analysis (see Miguel, 2016, for an overview). However, to the best of our knowledge there have been no attempts made to synthesize the findings in the form of a systematic review. We believe this is important to our understanding of verbal behavior development, and in offering a behavior analytic perspective on how children acquire the names of things. The importance of such an effort is further underlined given that naming improves some children’s learning speeds and facilitates complex behaviors such as reading and analogical responding (Eby et al., 2010; Meyer et al., 2019).

At the outset, we aimed to highlight the conceptual underpinnings of naming studies, the terminology that was adopted, and characterize whether the findings serve to accrue evidence for naming as an explanation for other behaviors such as categorization and equivalence, or if they focused on naming itself. In doing so, we discovered key differences in the methods used to test naming across studies. Thus, our systematic review aimed to identify studies that targeted naming and highlight (1) the methods that were used to test naming in terms of the stimuli, procedures and the criteria adopted; (2) the terminology adopted and the conceptual underpinnings of naming described in these studies; and (3) the methods used to train naming if it was found to be absent.

## Method

We undertook a systematic review of studies that conducted empirical investigations on naming. In particular, we were interested in the methods that were used to test naming, and those used to train naming if the behavior was found to be absent. We followed the *Preferred Reporting Items for Systematic Reviews and Meta-Analyses* (PRISMA) guidelines for the systematic review.

### Inclusion Criteria

We included articles that reported empirical data on naming. This meant that we included studies in which (1) listener behavior was trained and the emergence of speaker behavior was measured, or vice-versa; or (2) a naming exposure was provided and the emergence of either listener or speaker naming or both were measured. We also included studies only if they listed naming or bidirectional naming as a keyword, or used the naming theory (Horne & Lowe, 1996) to describe the study’s rationale or results. Studies were excluded from the review if they were theoretical articles or if they targeted intraverbal naming. We chose to exclude articles on intraverbal naming to focus on basic naming, i.e., *common bidirectional naming* as defined by Miguel (2016). In doing so, we are not suggesting that intraverbal naming is less important in terms of language development. However, intraverbal naming,^2^ almost by definition, can only emerge in the behavioral repertoire of a child once “basic” naming (i.e., relating an object with its name) has been established. At this stage, therefore, it seems important to focus our review on the most basic naming “unit,” but of course subsequent reviews might target intraverbal naming. If both common and intraverbal bidirectional naming were tested/trained in a study, we included such studies and only reported on common bidirectional naming. We excluded unpublished dissertations in our review.

### Search Procedure

We first conducted a database search to identify articles that met our inclusion criteria. We conducted searches on PubMed, Scopus, Web of Science, and ProQuest on April 11, 2022 and included articles that were published until that date. The search terms included “unidirectional naming OR bidirectional naming OR incidental naming OR listener naming OR speaker naming OR listener component of naming OR speaker component of naming OR unidirectional naming OR bidirectional naming” as well as a filter for the type of publication (i.e., peer-reviewed journal articles). We did not use any date range for the studies and did not include the term “naming” to avoid studies that used the term colloquially. The search terms varied slightly based on the constraints of the selected databases (see supplementary information for the complete search strategy used for each database).

Once eligible studies were identified, we searched their reference lists to identify additional articles. Finally, we conducted a citation search on Google Scholar to identify articles that cited the included studies to identify additional articles that met our inclusion criteria. We identified articles in English, Spanish and Brazilian Portuguese. The first author conducted the searches and the screening. An independent observer reviewed the full texts against the inclusion criteria. The agreement between reviewers was 100%.

### Outcome Measures and Data Extraction

The first author extracted information on the following areas from the eligible studies: participant characteristics, the type of naming targeted in the study (i.e., unidirectional naming/bidirectional naming/listener naming/speaker naming/incidental naming), the method used to test naming including the stimuli used, mastery criterion, type of naming exposure (if applicable), the method used to train naming (if applicable), and the conceptual underpinnings of the study (i.e., naming to explain equivalence/categorization or explaining naming itself). If the study participants were trained in naming and then administered equivalence/categorization tests, then we classified these studies as “using naming to explain equivalence/categorization.” If the study participants were trained to demonstrate naming, then the study was classified as “explaining naming itself.” We recognize that in the case of the former studies, it might be argued, perhaps in some cases, that the research was focused on both explaining naming *and* assessing its impact on equivalence/categorization. Making such a discrimination, in our view, seemed difficult, and thus it allowed for a more precise method for categorizing the studies based on whether they aimed to study naming in its own right *or* use naming to improve some other related performance (i.e., equivalence/categorization). The participant characteristics coded were the demographic variables including age, gender, and any diagnostic information.

An independent observer coded data from 35% of articles (16 included studies) and interobserver agreement was found to be 99.4%. In addition, all non-English articles were reviewed by the first author (using Google translate) and by a behavior analyst fluent in Spanish and Brazilian Portuguese. Interobserver agreement for the non-English articles was 100%.

## Results

**Fig. 1:**
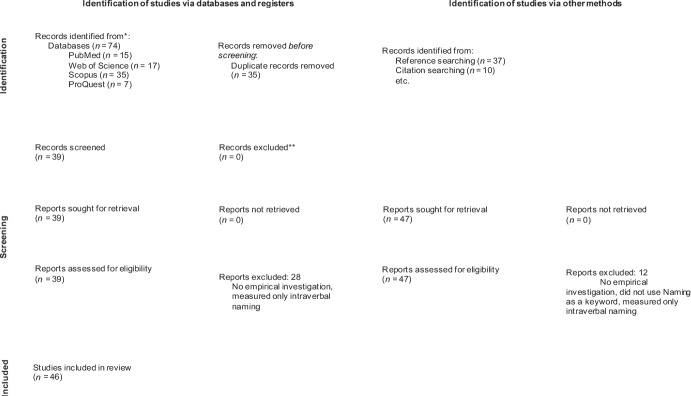
Reference Attrition Process

A total of 74 articles were identified through the database search. Following the removal of duplicates, 39 unique articles were included in the initial screening. Of these, 11 articles were deemed eligible for the review. The reference list search of these articles resulted in the screening of an additional 37 articles. Of these, 28 articles met our inclusion criteria. Finally, the citation search generated seven additional articles. Thus, a total of 46 articles were included in the current review. These 46 studies involved 56 experiments (i.e., some studies involved more than one experiment). Three experiments each from Petursdottir et al. (2020) and Cahill and Greer (2014), two experiments each from Miguel et al. (2008), Mahoney et al. (2011), Lowe et al. (2005), Pérez-González et al. (2011), Greer et al. (2011) and Greer and Du (2015), and only one experiment from Carr and Blackman (2001), Morgan et al. (2021), Cao and Greer (2019), Luciano et al. (2007) and Lowe et al. (2002) met inclusion criteria for the review. Forty-two of the included studies were reported in English, three in Brazilian Portuguese and one in Spanish. Figure 1 presents a summary of the reference attrition process. See Tables 1, 2 and 3 for a summarized display of the results described below.

**Table 1:** Terminology Used and Conceptual Underpinnings of Included Studies

Study	Terminology reported in the study	Classification based on Hawkins et al. (2018)	Conceptual underpinnings
Byrne et al. (2014)	Naming	Inc-BiN	Explain naming
Cahill & Greer (2014)	Naming	Inc-BiN	Explain naming
Cao and Greer (2019)	BiN	Inc-BiN	Explain naming
Carnerero & Pérez-González et al. (2014)	Naming	Inc-BiN	Explain naming
Carnerero and Pérez-González (2015)	Naming	Inc-BiN	Explain naming
Carnerero et al. (2019)	Naming (tact selection naming and pairing naming)	Inc-BiN	Explain naming
Carr and Blackman (2001)	Naming	Listener unidirectional incidental naming	Naming facilitates equivalence
da Silva Pereira et al. (2018)	BiN	Inc-BiN	Explain naming
Delfs et al. (2014)	Naming	Both listener and speaker unidirectional naming (BiN)	Explain naming
dos Santos and de Souza (2016)	Naming	Inc-BiN	Explain naming
Fiorile & Greer et al. (2007)	Naming	Inc-BiN	Explain naming
Gilic & Greer et al. (2011)	Naming	Inc-BiN	Explain naming
Greer and Du (2015)	Naming/Naming by exclusion	Speaker unidirectional naming	Explain naming
Greer et al. (2011)	Naming	Inc-BiN	Explain naming
Greer et al. (2007)	Naming	Inc-BiN	Explain naming
Greer et al. (2005)	Naming	Inc-BiN	Explain naming
Hawkins et al. (2007)	Naming	Inc-BiN	Explain naming
Hawkins et al. (2009)	Naming	Inc-BiN	Explain naming
Horne et al. (2006)	Naming	Speaker unidirectional naming	Naming explains categorization
Horne et al. (2004)	Naming	Speaker unidirectional naming	Naming explains categorization
Hotchkiss and Fienup (2020)	BiN	Inc-BiN	Explain naming
Kobari-Wright and Miguel (2014)	Naming	Speaker unidirectional naming	Naming explains categorization
Lee et al. (2021)	BiN	Inc-BiN	Explain naming
Lee et al. (2015)	Naming	Speaker unidirectional naming	Naming explains categorization
Lobato and De Souza (2020)	BiN	Inc-BiN	Explain naming
Longano and Greer (2014)	Naming	Inc-BiN	Explain naming
Lowe et al. (2002)	Naming	Listener unidirectional naming	Naming explains categorization
Lowe et al. (2005)	Naming	Listener unidirectional naming	Naming explains categorization
Luciano et al. (2007)	Naming (Vocal and nonvocal naming)	Inc-BiN	Explain naming
Mahoney et al. (2011)	Naming	Listener unidirectional naming	Naming explains categorization
Miguel and Kobari-Wright (2013)	Naming	Listener unidirectional naming	Naming explains categorization
Miguel et al. (2008)	Naming	Both listener and speaker unidirectional naming (BiN)	Naming explains categorization
Miller et al. (2021)	BiN	Speaker unidirectional naming	Explain naming
Morgan et al. (2021)	BiN	Inc-BiN	Explain naming
Olaff and Holth (2020)	BiN	Inc-BiN	Explain naming
Olaff et al. (2017)	Naming	Inc-BiN	Explain naming
Pérez-González et al. (2014)	Naming	All three: listener, speaker unidirectional, and Inc-BiN	Explain naming
Pérez-González et al. (2011)	Naming	All three: listener, speaker unidirectional, and Inc-BiN	Explain naming
Petursdottir et al. (2020)	BiN	Speaker unidirectional naming	Explain naming
Ribeiro and Miguel (2020)	BiN	Listener unidirectional naming	Naming explains categorization
Ribeiro et al. (2018)	BiN	Listener unidirectional naming	Naming explains categorization
Rosales et al. (2011)	Naming	Speaker unidirectional naming	Explain naming
Rothstein and Gautreaux (2007)	Naming	Inc-BiN	Explain naming
Sivaraman et al. (2021)	BiN	Inc-BiN	Explain naming
Speckman-Collins et al. (2007)	Naming	Listener incidental unidirectional naming	Explain naming
Sprinkle and Miguel (2012)	Naming	Both listener and speaker unidirectional naming (BiN)	Naming explains categorization

**Table 2:** Stimuli and Methods Used to Test Naming

Study	Stimuli per set	Type of naming exposure used	Stimulus presentation method	Number of exposures per stimulus	Number and types of naming trials per stimulus	Criterion for naming
Byrne et al. (2014)	3	Stimulus pairing	Simultaneous	15	3 listener and tact trials each	88.9% across three blocks
Cahill and Greer (2014)	3	MTS	Simultaneous	6		
Cao and Greer (2019)	5	MTS	Simultaneous	8	4 listener and tact trials each	80%
Carnerero & Pérez-González et al. (2014)	5	Stimulus pairing	Simultaneous	4	4 listener and tact trials each	90%
Carnerero and Pérez-González (2015)	4	Stimulus pairing	Simultaneous	2	2 listener, tact and intraverbal trials each	87.5%
Carnerero et al. (2019)	4	Stimulus pairing	Simultaneous	2	2 listener and tact trials each	87.5%
Carr and Blackman (2001)	6; each given one of two names	Stimulus pairing	Simultaneous	Varied based on mastery criterion	Varying number of trials	
da Silva Pereira et al. (2018)	5	MTS	Simultaneous	5	3 listener and tact trials each	
Delfs et al. (2014)	2	NA	NA	NA	Unclear	75% for three participants; 80% for one participant
dos Santos and de Souza (2016)	3	MTS	Simultaneous	Varied based on mastery criterion	6 listener and tact trials each	88.9%
Fiorile & Greer et al. (2007)	3	NA	NA	NA	2 listener, tact and impure tact trials each	94.4% for listener, 100% for all speaker responses
Gilic & Greer et al. (2011)	3	MTS	Simultaneous	4	4 listener and tact trials each	80%
Greer and Du (2015)	5	Exclusion learning trials	Simultaneous	4	2 listener and intraverbal tact trials each	80%
Greer et al. (2011)	5	MTS	Simultaneous	Varied based on mastery criterion	4 listener, tact and intraverbal tact trials each	80%
Greer et al. (2007)	5	MTS	Simultaneous	Varied based on mastery criterion	4 listener, tact and impure tact trials each	80%
Greer et al. (2005)	5	MTS	Simultaneous	4	4 listener, tact and impure tact trials each	80%
Hawkins et al. (2007)	5	MTS	Simultaneous	Varied based on mastery criterion	4 listener, tact and impure tact trials each	80%
Hawkins et al. (2009)	4	MTS	Simultaneous	Varied based on mastery criterion	4 listener, tact and impure tact trials each	90%
Horne et al. (2006)	6; each given one of two names	NA	NA	NA	4 tact trials per stimulus	75%
Horne et al. (2004)	6; each given one of two names	NA	NA	NA	3 tact trials per stimulus	88.9%
Hotchkiss and Fienup (2020)	5	MTS	Simultaneous	4	2 listener, tact and intraverbal tact trials each	80%
Kobari-Wright and Miguel (2014)	9; each given one of three names	NA	NA	NA	4 listener and tact trials each	89%
Lee et al. (2021)	4	MTS	Simultaneous	Varied based on mastery criterion	5 listener, tact and intraverbal tact trials each	80%
Lee et al. (2015)	9; each given one of three names	NA	NA	NA	4 listener and tact trials each	89%
Lobato and De Souza (2020)	3	MTS	Simultaneous	Varied based on mastery criterion	3 listener and tact trials each	78%
Longano and Greer (2014)	4	MTS	Simultaneous	Varied based on mastery criterion	5 listener, tact and intraverbal tact trials each	80%
Lowe et al. (2002)	6; each given one of two names	NA	NA	NA	4 listener trials per stimulus	75%
Lowe et al. (2005)	6; each given one of two names	NA	NA	NA	2 listener trials per stimulus	69.4%
Luciano et al. (2007)	1	Stimulus pairing	Simultaneous	3	Varying number of trials	NA
Mahoney et al. (2011)	6; each given one of two motor responses	NA	NA	NA	2 listener trials per stimulus	NA
Miguel and Kobari-Wright (2013)	9; each given one of three names	NA	NA	NA	4 listener and tact trials each	89%
Miguel et al. (2008)	6; each given one of two names	NA	NA	NA	At least 12 listener trials in all (6 per assigned name)	NA
Miller et al. (2021)	4	Stimulus pairing	Simultaneous	4	2 tact trials per stimulus	87.5%
Morgan et al. (2021)	5	MTS	Simultaneous	4	4 listener and tact trials each	80%
Olaff and Holth (2020)	4	Stimulus pairing	Simultaneous	5	5 listener, tact and manded tact trials each	80%
Olaff et al. (2017)	5	MTS	Simultaneous	Varied based on mastery criterion	4 listener, tact and impure tact trials each	70%
Pérez-González et al. (2014)	3	Stimulus pairing	Simultaneous	6	4 or 6 listener and tact trials each	83.3%
Pérez-González et al. (2011)	3	Stimulus pairing (Experiments 2A and 2B)	Simultaneous	10	2 listener, tact trials per stimulus	NA
Petursdottir et al. (2020)	4	Stimulus pairing	Non-simultaneous (Word-first/Image-first)	5	2 listener, 1 tact trial	NA
Ribeiro and Miguel (2020)	9; each given one of three names	NA	NA	NA	4 listener and tact trials each	89%
Ribeiro et al. (2018)	9; each given one of three names	NA	NA	NA	4 listener and tact trials each	89%
Rosales et al. (2011)	4	NA	NA	NA	2 listener and tact trials each	87.5%
Rothstein and Gautreaux (2007)	3	MTS	Simultaneous	Not reported	Not reported	90%
Sivaraman et al. (2021)	1	Stimulus pairing	Non-simultaneous	3	3 listener, 1 speaker trial per stimulus	NA
Speckman-Collins et al. (2007)	4	MTS	Simultaneous	Varied based on mastery criterion	5 listener and tact trials each	Not reported
Sprinkle and Miguel (2012)	3	NA	NA	NA	3 listener and tact trials each	NA

**Table 3:** Methods Used to Train Naming

Study	Methods used	Participants trained successfully
Byrne et al. (2014)	Stimulus pairing observation procedure with multiple exemplar instruction	All three participants met criterion with MEI sets; only one participant met criterion with novel set
Cahill and Greer (2014)	Multiple exemplar instruction	All four participants met criterion for MEI and demonstrated listener and speaker naming responses
Cao and Greer (2019)	Echoic training	Training was effective in establishing BiN in Chinese with familiar visual stimuli for all participants and BiN in Chinese with nonfamiliar visual stimuli for five participants
Carnerero & Pérez-González et al. (2014)	Picture-name pairing	All four participants acquired untrained listener and speaker responses following the intervention
Carnerero and Pérez-González (2015)	Pairing procedure	All 12 participants acquired tacts after the pairing procedure
Carnerero et al. (2019)	Pairing of names with sounds	All eight participants demonstrated selection (listener) responses, seven participants demonstrated speaker (tact) responses
Carr and Blackman (2001)	No intervention	-
da Silva Pereira et al. (2018)	Multiple exemplar instruction	The participant demonstrated bidirectional naming
Delfs et al. (2014)	Trained tacts and probed for listener (and vice-versa) using prompts and programmed reinforcement	Tact training was either equally or more efficient than listener training for all four participants
dos Santos and de Souza (2016)	Multiple exemplar instruction	All participants met criteria for MEI. Two out of four participants demonstrated bidirectional naming with novel stimuli
Fiorile & Greer et al. (2007)	Multiple exemplar instruction	Naming emerged for all four participants following MEI
Gilic & Greer et al. (2011)	Multiple exemplar instruction	All participants met criteria for MEI. Seven out of eight children demonstrated naming with untrained stimuli
Greer and Du (2015)	Exclusion multiple exemplar instruction	All 8 participants acquired naming by exclusion following training; 1 participant in the control group also acquired naming by exclusion
Greer et al. (2011)	Individual sessions of listener, match-to-sample, tact and intraverbal training; Multiple exemplar instruction	All four participants demonstrated naming following the intervention
Greer et al. (2007)	Single exemplar instruction (SEI) or multiple exemplar instruction	Naming emerged for all four participants who received MEI; Naming did not emerge for participants receiving SEI
Greer et al. (2005)	Multiple exemplar instruction	Untaught speaker responses emerged at 60%–85% for two participants, and 40%–70% for one participant
Hawkins et al. (2007)	Multiple exemplar instruction	One participant out of three demonstrated naming after the intervention
Hawkins et al. (2009)	Multiple exemplar instruction	All three participants acquired naming postintervention
Horne et al. (2006)	Listener behavior training	Ten out of fourteen participants passed the tact test
Horne et al. (2004)	Listener behavior training	Two out of nine participants passed the tact test
Hotchkiss and Fienup (2020)	Intensive tact instruction with two different intensities	Participants with unidirectional naming demonstrated BiN under both intensities; participants who did not show naming prior to the study did not acquire BiN under either intensity of the intervention
Kobari-Wright and Miguel (2014)	Trained listener using prompts and programmed reinforcement and probed for tacts	Three participants demonstrated emergence of untrained tacts and one participant did not do so
Lee et al. (2021)	Multiple exemplar instruction	All three participants demonstrated improvements in naming after the intervention
Lee et al. (2015)	Trained listener using prompts and programmed reinforcement and probed for tacts	Two out of four participants demonstrated untrained speaker responses
Lobato and De Souza (2020)	Multiple exemplar instruction or stimulus pairing observation procedure	One participant demonstrated naming after both MEI and SPOP. Two participants only demonstrated listener component (not speaker) of naming after SPOP
Longano and Greer (2014)	Conditioning of visual and auditory stimuli as reinforcers	All three participants showed improvements in naming responses postintervention
Lowe et al. (2002)	Vocal tact training	All participants demonstrated untrained listener behavior
Lowe et al. (2005)	Vocal tact training	All participants demonstrated untrained listener behavior
Luciano et al. (2007)	Multiple exemplar training	The participant demonstrated untrained receptive and speaker responses posttraining
Mahoney et al. (2011)	Motor response tact training	Three out of five participants passed listener tests after motor response training
Miguel and Kobari-Wright (2013)	Trained speaker responses using prompts and programmed reinforcement and probed for listener responses	Both participants emitted untrained listener behavior
Miguel et al. (2008)	Tact training and listener training with prompting and programmed reinforcement	All four participants demonstrated some untrained listener and speaker behavior after tact and listener training respectively
Miller et al. (2021)	No intervention	-
Morgan et al. (2021)	No intervention	-
Olaff and Holth (2020)	Sequential operant instruction	Two out of four participants demonstrated bidirectional naming after the intervention
Olaff et al. (2017)	Multiple response exemplar training	All three participants showed improvements in naming post-training
Pérez-González et al. (2014)	Tact/Selection training and pairing procedure	Five out of seven participants showed emergence of selection responses and tacts
Pérez-González et al. (2011)	Tact training and listener training with prompting and programmed reinforcement; pairing procedure	All five participants demonstrated naming
Petursdottir et al. (2020)	No intervention	-
Ribeiro and Miguel (2020)	Speaker training with prompting and programmed reinforcement	Both participants emitted untrained listener behavior
Ribeiro et al. (2018)	Speaker training with prompting and programmed reinforcement	All four participants emitted untrained listener behavior
Rosales et al. (2011)	Listener training or Multiple exemplar training	All four participants demonstrated improvements in untrained tact responses post-MET
Rothstein and Gautreaux (2007)	Peer-yoked contingency	All three participants demonstrated improvements in naming following training
Sivaraman et al. (2021)	Multiple exemplar training	All participants who underwent training demonstrated listener responses, only one participant demonstrated both listener and speaker responses
Speckman-Collins et al. (2007)	Generalized auditory matching	Both participants showed an improvement in the listener component of naming posttraining
Sprinkle and Miguel (2012)	Speaker training and listener training with prompting and programmed reinforcement	Speaker training always resulted in the emergence of untrained listener behavior

### Demographic Characteristics

A total of 319 participants were reported across all 46 studies included in the review. Of these, there were 154 male and 108 female participants. Fourteen studies (e.g., Carr & Blackman, 2001; Greer et al., 2011; Miguel et al., 2008; Miller et al., 2021; Rosales et al., 2011) did not report the gender of the participants. Forty-three studies involved children as participants and they included a total of 290 children (145 males and 94 females) and the remaining three studies (Carnerero et al., 2019; Carnerero & Pérez-González, 2015; Carr & Blackman, 2001) involved 29 adults (9 males and 14 females). Twenty-nine of the studies included children with a developmental disability (autism spectrum disorder, pervasive developmental disorder, cerebral palsy, specific language impairment, other health impairment, multiple disabilities) or a developmental delay. The average age of the child participants was 5.7 years (*SD* = 3.18) and that of the adult participants was 25.6 years (*SD* = 5.13). Two studies (Carr & Blackman, 2001; Rosales et al., 2011) did not report the ages of all the participants. Six studies reported on a total of 44 children under the age of 3 years.

### Naming Terminology Used

Twelve of the included studies used the term *bidirectional naming* to describe the bidirectional relation between the listener and speaker behaviors. That is, studies used this term when (1) listener behavior was trained and speaker behavior emerged without any training or programmed reinforcement; or (2) both listener and speaker behavior emerged following a naming experience without programmed consequences. The remaining 34 studies simply referred to the behavior as *naming* (or as *Naming*)*.* It is important to note that 12 of the 13 studies published from 2018 onwards adopted the phrase *bidirectional naming* following the terminology put forth by Miguel (2016).

Furthermore, 15 studies described the *incidental* learning of names in which individuals acquired both the listener and speaker responses without direct instruction or programmed consequences but used the term *bidirectional naming* or *Naming* to refer to this behavior. Other terminology such as *pairing naming* were also used to describe incidental naming in some studies (e.g., Carnerero & Pérez-González, 2014).

We used the Hawkins et al. (2018) classification to highlight the specific type of naming that was tested in each study. The majority of studies included in the review tested incidental bidirectional naming (Inc-BiN; the emergence of both listener and speaker behavior without programmed consequences following a naming experience). However, as mentioned earlier, these studies did not describe the behavior as Inc-BiN. In particular, 25 studies tested Inc-BiN, and 2 studies tested listener incidental unidirectional naming (i.e., emergence of listener behavior following a naming experience). Eight studies tested speaker unidirectional naming (i.e., emergence of speaker behavior following listener training); six studies tested listener unidirectional naming (i.e., emergence of listener behavior following speaker training); three studies tested bidirectional naming (i.e., emergence of listener following speaker training, and emergence of speaker behavior following listener training with a novel set each); and two studies tested both bidirectional naming and incidental bidirectional naming.

### Methods to Test Naming

#### Stimuli Used

Thirty studies used two-dimensional stimuli (i.e., printed pictures and images on a computer screen), nine studies used three-dimensional stimuli (e.g., objects, wooden shapes), and four studies used both two- and three-dimensional stimuli. Two studies (Carnerero et al., 2019; Carnerero & Pérez-González, 2015) used sounds of musical instruments, and one study (Cahill & Greer, 2014) used actions as stimuli. All six studies with participants under the age of 3 years used three-dimensional stimuli. Twelve studies used abstract stimuli to test naming. The abstract stimuli included computer-generated shapes and wooden shapes that were assigned nonsense names. Twenty-eight studies used commonly found stimuli including types of dogs, birds, dinosaurs, household items, cooking utensils, pasta, gemstones, monuments, famous personalities, professions, Greek letters, Chinese symbols etc. Four studies used a combination of abstract and commonly found stimuli. One study (Hawkins et al., 2007) did not report the type of stimuli used to test naming. Overall, the majority of studies used commonly occurring items as stimuli, there was wide variation in the types of stimuli that were used, and some studies used items that belonged to the same category (e.g., gemstones) whereas others did not do so.

#### Number of Stimuli per Set

Thirty-four studies conducted naming tests in which each stimulus was given a unique name. Two of these studies (Luciano et al., 2007; Sivaraman et al., 2021) used only one stimulus at a time during the naming test. The remaining 32 studies used between two and five stimuli at a time to test naming, with 1 study (Delfs et al., 2014) using two stimuli per test, 10 studies each using three and four stimuli per test, and 11 studies using five stimuli per test. Twelve other studies conducted naming tests in which sets of three stimuli were given the same name, and these studies typically involved either two or three sets. That is, an overwhelming majority of studies used a minimum of three stimuli per administration of the naming test. Only two studies tested naming with one stimulus at a time.

#### Naming Experience

Studies reported using three types of naming experiences prior to administering the naming test trials. These naming experiences served to pair the target stimulus with its name and involved either: (1) match-to-sample trials; (2) stimulus-stimulus pairing; or (3) naming-by-exclusion learning opportunities. A match-to-sample (MTS) type naming experience involved identity-matching trials. Researchers held up the target stimulus, presented a comparison array consisting of either two or three stimuli, and provided the instruction “Match [stimulus name].” Participants were required to match the target stimulus with the correct comparison while hearing its name. Stimulus–stimulus pairing involved the researcher presenting the target stimulus and stating its name as soon as the child made visual (or auditory) contact with the target. Eighteen studies reported using MTS naming experiences and 11 studies used stimulus–stimulus pairing. One study (Greer & Du, 2015) reported using a naming-by-exclusion learning opportunity as the naming experience. During these trials, the researcher presented an array comprised of two known items and one item unknown to the child (these items were previously confirmed to be *known* and *unknown* to the participants). Participants were then instructed to “point to [unknown item name],” and these trials served as naming experiences. The remaining studies did not provide a naming experience during the naming test. Overall, MTS trials were the most commonly used method to provide a naming experience, despite having the least ecological validity in that young children typically encounter novel objects during interactions with a caregiver as described in the introduction.

Two studies that used the stimulus pairing procedure reported variations in the method used for the presentation. Petursdottir et al. (2020) used two sequential arrangements that involved either a word-first or an image-first condition. In the word-first condition, the word was presented first followed by the presentation of the image, and in the image-first condition, the image appeared first and the word was presented after the image disappeared from the screen. Sivaraman et al. (2021) used a nonsimultaneous presentation format wherein researchers presented the target visual stimulus, and upon visual contact, covered it with a cloth before stating its name. This type of nonsimultaneous presentation seemed to disrupt naming responses in young children.

Furthermore, the studies that conducted a naming experience provided a predetermined number of exposures per target stimulus. Whereas 11 of the studies that used the MTS trials set a mastery criterion for participants to meet prior to administering naming test trials, 6 other studies that used the MTS trials and 10 studies that used the stimulus pairing procedure provided a specific number of exposures per stimulus. Six of these studies (e.g., Carnerero & Pérez-González, 2014; Gilic & Greer, 2011) used 4 exposures per stimulus, three studies conducted 5 exposures per stimulus, two studies each conducted 2 and 3 exposures per stimulus respectively, and one study each conducted 6, 8, and 15 exposures per stimulus. Greer and Du (2015) also conducted four exposures per stimulus using the naming-by-exclusion trials. In the studies that set a mastery criterion, participants received a varying number of exposures per stimulus based on the number of sessions they took to meet the criterion. The criteria ranged between 80% and 100% correct matching responses for one or two sessions. There was wide variation in the number of exposures that were provided per stimulus across the included studies.

#### Test Trials

Test trials for naming were comprised of listener, tact and/or intraverbal trials. Listener trials involved the researcher presenting a name followed by an array of two or three comparison stimuli. Researchers typically ensured that the trial was presented only when the participant oriented towards the stimulus which was ensured by waiting for their visual attention, or providing the instruction “Look” or having the participant touch the screen. Luciano et al. (2007) described listener trials as receptive symmetry trials. During a speaker trial, a researcher held up a stimulus and waited between 2 s and 5 s for the participant to respond. In some instances, the researcher asked, “What is this?” while holding up the stimulus. Some researchers referred to these trials as tact trials (e.g., Carnerero & Pérez-González, 2015) or speaker trials (Sivaraman et al., 2021), whereas others referred to these as intraverbal tact trials (e.g., Hotchkiss & Fienup, 2019) or as impure tact trials (e.g., Fiorile & Greer, 2007; Greer et al., 2005; Hawkins et al., 2007; Olaff et al., 2017). Although there is some inconsistency in the terms used across studies, they all seem to be reporting on functionally similar performances.

There were variations in the number of trials that were conducted across studies. Researchers conducted between one and six trials per stimulus per operant during the naming test. In particular, 18 studies conducted four trials (i.e., listener trials or tact trials or both) for each stimulus (e.g., Cao & Greer, 2019; Horne et al., 2006; Miguel & Kobari-Wright, 2013; Olaff et al., 2017; Pérez-González et al., 2011). Ten studies conducted two trials per stimulus used (e.g., Greer & Du, 2015; Hotchkiss & Fienup, 2020; Rosales et al., 2011), four studies each conducted three trials per stimulus (Byrne et al., 2014; Horne et al., 2004; Lobato & Souza, 2020; Pereira et al., 2018), and five studies conducted five trials per stimulus (e.g., Lee et al., 2021; Speckman-Collins et al., 2007). Dos Santos and de Souza (2016) conducted six listener and tact trials for each stimulus, whereas Petursdottir et al. (2020) and Sivaraman et al. (2021) conducted one tact (speaker) trial per stimulus used during the naming test. The remaining studies conducted a varying number of trials per stimulus (e.g., Luciano et al., 2007). Overall, all included studies used listener and/or speaker trials to demonstrate the presence/absence of naming, but there seemed to be several variations in the number of trials that were used for the naming test.

#### Mastery Criteria

There were variations reported in the mastery criteria for naming used across studies and these criteria ranged between 70% and 90%, with the most commonly reported criterion to confirm the presence/emergence of naming being 80%.

To summarize, the testing procedures used across studies revealed wide variations in the type of stimuli used, the number of exposures used during the naming experience, the type and number of trials and mastery criterion used to demonstrate naming. A majority of the studies used sets with multiple stimuli, and MTS trials as naming experiences.

### Methods Used to Train Naming

A number of procedures were tested across studies for their efficacy and/or utility in inducing naming responses in the study participants. These procedures included multiple exemplar training (e.g., Luciano et al., 2007; Rosales et al., 2011; Sivaraman et al., 2021), multiple exemplar instruction (e.g., Greer et al., 2005; Lee et al., 2021), echoic training (e.g., Cao & Greer, 2019), stimulus pairing observation procedure (Byrne et al., 2014; Carnerero & Pérez-González, 2014), conditioning of social reinforcers (e.g., Olaff & Holth, 2020), conditioning of pictures and sounds as reinforcers (e.g., Longano & Greer, 2014), and intensive tact training (Hotchkiss & Fienup, 2020). Multiple exemplar training typically involved training across one response type (e.g., listener responses) whereas multiple exemplar instruction involved training across response types (e.g., listener, tact, echoic, and match-to-sample trials trained within each block). Echoic training sessions were comprised of participants correctly pronouncing target phonemes with point-to-point correspondence immediately after the researcher had enunciated the sounds. The conditioning procedure for social reinforcement involved participants opening a box contingent on teacher-presented praise, smiles, and nods. Access to the box was blocked in the absence of teacher-presented social stimuli. During intensive tact training, participants were taught an additional 50 (or 100) tacts besides their daily curriculum. The stimulus pairing observation procedure involved researchers presenting the target stimulus while dictating its name and these trials were repeated a predetermined number of times. Some studies (e.g., Delfs et al., 2014; Kobari-Wright & Miguel, 2014) trained either the listener or speaker response, and used prompting (i.e., vocal prompting, gestural prompting), error correction and programmed reinforcement during the intervention. See Table 3 for an overview of the training strategies and results reported across studies.

The aforementioned list of training procedures is by no means comprehensive. There were multiple procedural variations reported across studies using the same training procedure. For instance, Carnerero and Pérez-González et al. (2014) used the stimulus pairing observation procedure (described as picture-name pairing by the authors) in which instructional sessions were comprised of 20-trial blocks wherein each stimulus in the set was paired with its name five times. Byrne et al. (2014) also used the same procedure, but conducted 45-trial instructional sessions in which each stimulus was paired with its name 15 times. We noted such variations across several included studies.

In terms of the efficacy of these intervention procedures, nearly all studies reported positive outcomes for a majority of the participants. Each study reported improvements in listener and/or speaker behavior in at least one participant, and 27 studies reported that all participants met the mastery criterion set for the training. Overall, a wide range of intervention procedures were tested, and although there were variations in the implementation of these procedures, they seemed effective at training the targeted naming responses in the participants.

### Conceptual Underpinnings

As noted previously, we classified the studies included in the review into one of two groups depending on whether they used naming to explain equivalence or categorization, or whether they focused on how naming itself emerges. All studies fell into one of these two categories.

#### Naming to Explain Equivalence and Categorization

Thirteen of the included studies (see Table 1) used naming to explain equivalence or categorization. In particular, 11 studies used naming to explain categorization and 2 studies (Carr & Blackman, 2001; Sprinkle & Miguel, 2012) used naming to explain equivalence class formation. All of the categorization studies typically used a set of six (or nine) stimuli, and each stimulus was given one of two (or three) category names respectively. For example, Horne et al. (2006) used a set of six stimuli and each stimulus was named either “zog” or “vek.” Likewise, Miguel and Kobari-Wright (2013) used nine picture stimuli which belonged to one of three categories of dogs (i.e., hound dog, toy dog, and work dog). The studies that tested for equivalence class formation used three-member equivalence classes which either involved Greek symbols (Carr & Blackman, 2001) or classes comprised of a picture, a printed word, and a spoken word (Sprinkle & Miguel, 2012).

All of these studies either trained listener responses and tested participants for the emergence of speaker responses, or vice-versa. In addition, the studies tested for the emergence of categorization or equivalence class formation. Categorization tests were conducted using a match-to-sample format. For example, in Miguel and Kobari-Wright (2013) the researchers presented a picture of one of the stimuli as the sample and a three-stimulus comparison array during the categorization trials. A correct response involved selecting the stimulus that belonged to the same category as the sample. Tests for equivalence class formation involved training two relations (e.g., A1–B1 and B1–C1) and testing for transitivity (e.g., C1–A1).

#### Explaining Naming

Thirty-three studies aimed to explain the emergence of naming. Twenty-nine of these studies tested procedures to induce naming when it was found to be missing in a child’s repertoire. Morgan et al. (2021) evaluated the relationship between bidirectional naming and arbitrarily applicable relational responding. Petursdottir et al. (2020) tested the impact of variations in the presentation of the naming experience (i.e., sequential variations in the presentation of the image and the name) on naming responses. Miller et al. (2021) evaluated the impact of blocking the echoic response on the emergence of speaker naming and Carnerero et al. (2019) tested the impact of probing listener behavior first on the emergence of tacts and intraverbal responses.

## Discussion

We conducted a systematic review of the naming literature to characterize the terminology adopted, the conceptual underpinnings, and the methods used to test and train naming. We identified 46 studies that met our inclusion criteria, and found that approximately one third of these studies attempted to offer evidence that naming facilitated some other type of behavior (i.e., categorization or stimulus equivalence). Most of the remaining studies attempted to train naming in individuals when the behavior was found to be absent. All studies published from 2018 used the term *common bidirectional naming* to refer to the behavior as proposed by Miguel (2016). We found wide variation in the methods used to test naming. There were differences in the types of stimuli, the naming experiences used to pair objects with their spoken names, and the number and types of trials used to confirm the presence of naming. We found similar variations in the methods adopted to train naming.

Some of the results highlighted above warrant additional discussion. First, we observed that 44 of the 46 studies used more than one stimulus at a time to test naming. One explanation for the use of multiple stimuli could stem from the early history of naming research. In particular, the initial empirical studies aimed to show that naming facilitated equivalence, and it seems possible that using sets with multiple stimuli was a practice adopted from equivalence research (i.e., when participants were trained to name each of the stimuli in two or more equivalence classes). In addition, given that a majority of the participants in the included studies were over the age of 3 years, multiple stimuli may have been needed to avoid ceiling effects. Indeed, we are only speculating here on why multiple stimuli may have been used, nevertheless it seems important to identify the variables involved in learning to name one stimulus at a time before testing participants with multiple stimuli. It is important to note that we observed that the two studies that involved some of the youngest participants (Luciano et al., 2007; Sivaraman et al., 2021) used one stimulus at a time to test naming. Conducting research with infants and toddlers such as those in the studies above offers an important means to study the environmental conditions under which naming first emerges and gets established as a higher-order operant. We recognize that there are challenges associated with recruiting infants and keeping them engaged during experimental sessions. Nevertheless, such research seems crucial in advancing our understanding of the ontogenic history that establishes even the most basic of naming repertoires.

Only two of the included studies involved conditions where the object (or picture) and its name were not presented together. Sivaraman et al. (2021) argued that a derived bidirectional relation need not be invoked to explain the emergent naming responses when objects and their names are presented simultaneously. That is, when objects and their names are presented contemporaneously, the child sees object-hears name and also hears name-sees object at the same time. In such situations, it could be argued that the emergent listener (and/or speaker responses, assuming an echoic repertoire) are trained directly. On the other hand, when objects and their names are presented sequentially and nonsimultaneously, then the emergent responses would seemingly require derived (bidirectional) relational responding.^3^ In effect, the rationale for adopting nonsimultaneous presentations of objects and their names is to remove any possible training that may occur during the naming test. Psychologists have previously demonstrated that temporal synchrony between objects and their names may be essential, at least initially, for infants learning names. Over the course of the second year of life, young children seemed to relate objects with their spoken names even when they were presented with a brief delay (Gogate & Bahrick, 1998). The operant contingencies that facilitate such performance clearly need further investigation.

As outlined in the introduction, subsequent to Horne and Lowe’s (1996) seminal paper, new terminology to describe naming and its different types have been suggested in the literature by Miguel (2016) and Hawkins et al. (2018). However, our findings indicate that the adoption of these terminologies is mixed. In particular, more than half of the studies included in the review (including eight studies published after 2018) focused on what was described as *incidental bidirectional naming* by Hawkins et al.—that is, the emergence of listener and speaker responses to a set of stimuli without direct reinforcement, or instruction, for either of these responses. Although all of these studies outline the incidental learning of names, they simply used the term *bidirectional naming* to describe the behavior. On balance, nearly all studies published from 2018 onwards adopted the phrase *bidirectional naming* following the terminology put forth by Miguel (2016). We are not suggesting that all researchers studying naming adopt the taxonomy put forth by Hawkins et al., but the distinction between common bidirectional naming and incidental bidirectional naming has been described as being crucial (Greer & Ross, 2008). Although the former accounts for the emergence of untrained speaker responses when listener responses are trained (or vice-versa), the latter accounts for the untrained emergence of both topographies of responses. If such a distinction between *common* bidirectional naming and *incidental* bidirectional naming is indeed important, future research should aim to offer more precise descriptions of the type of naming being tested/trained.

Nearly two thirds of the studies that we included provided some type of naming experience (i.e., an event during which an object is paired with its name), and a majority of these studies used match-to-sample trials for the naming experience. In the natural environment, children have been shown to encounter novel objects or pictures when caregivers present these stimuli during play, book-reading, or other social interactions (Hart & Risley, 1995, 1999). Although some prior research on naming has been conducted in such naturalistic contexts (see, for example, Carey & Bartlett, 1978), more research simulating these naturalistic encounters of novel words may be required. Studies that are ecologically valid and are representative of real-life contexts in which children engage in naming responses would seem to be particularly relevant in research on the development of verbal behavior.

The establishment of verbal behavior, naming in particular, has received much attention within the field of behavior analysis. Previous research has shown that establishing bidirectional naming facilitates children’s reading, writing, spelling, and problem solving (Eby et al., 2010; Meyer et al., 2019). Thus, researchers have described naming as an important skill to be established during language development (Greer et al., 2017; Miguel, 2018; Miguel & Petursdottir, 2009). In the current review, we highlighted procedures that have been used to train naming across the included studies. These studies reported on a variety of successful procedures including multiple exemplar training, multiple exemplar instruction, conditioning of social reinforcers, echoic training, a stimulus-pairing observation procedure and others. Although there were procedural variations reported in these studies, overall, they seemed effective at establishing naming in children who did not demonstrate the behavior prior to the commencement of the study. Nevertheless, further work is necessary to translate these findings into a comprehensive tool for practitioners to consult when designing intervention procedures.

A first step towards assessing naming intervention effects across published studies could involve characterizing conditions (e.g., in terms of participants, study characteristics, etc.) under which a desired effect was present or not present (see, for example, Manolov et al., 2022, for a recent tutorial on assessing intervention effects). It could be useful for a future study to map out the efficacy of a naming intervention to a participant’s baseline behavioral repertoire to identify *what* works for *whom*. Such a summary of intervention effects seems like a logical step towards organizing the wealth of research that has been published on this topic.

There are some limitations to the current review that warrant consideration. First, we did not include studies on intraverbal naming and chose to focus on the basic naming repertoire. It is possible that including studies on intraverbal naming might have raised other issues concerning the terminology that have evolved in the study of naming and related verbal behaviors. Next, we did not compare the efficacy of the training procedures used across studies to establish naming. This seems like an important area for future research. In addition, we did not conduct a quality assessment to analyze the strength of evidence contained within the included studies. Despite these limitations, we feel that the current review highlights critical issues to be considered for future research on naming.

Several areas for future research have been highlighted above. Our recommendations include conducting studies with infants and testing naming with one stimulus at a time to gain insight into the first instances of naming, investigating differences in naming performance when objects and their names are presented simultaneously and nonsimultaneously. We also recommend that more studies provide naming experiences that are ecologically valid by designing experiments where children encounter novel words during play time or while reading a book. Greater consistency on the methods used to test naming in terms of the types of stimuli used and mastery criteria is warranted. It remains unclear if we can achieve consensus on the nomenclature or testing strategies for naming, but at the very least, it seems important to generate a list of focal aspects for empirical assessments of naming. Finally, future research could aim to characterize the intervention effects associated with strategies that have been used to train naming.

Overall, our review highlighted that there exists a rich empirical dataset on testing and training naming within behavior analysis. In particular, we found key variations in the terminology adopted, the testing stimuli and types of trials and the mastery criteria used to confirm the presence of naming (or lack thereof). This conclusion may be nuanced by differences in the sub-types of naming that have been identified (e.g., unidirectional naming, bidirectional naming) and the analytic purpose of the research (e.g., studying naming per se versus studying its impact on other behaviors, such as equivalence/categorization). Nevertheless, we believe that it calls for greater collaboration and cooperation between researchers studying naming. Although complex and challenging, such collaborations seem essential to highlight points of overlap and differences between the diverse conceptual perspectives within naming research. Such an attempt will greatly benefit the advancement of a behavior-analytic account of naming and language development, in general, while also bringing enormous rewards to students and practitioners of behavior analysis. We hope that the current study ultimately serves to inspire efforts in this direction.

## Data Availability

The data associated with this manuscript are available from the corresponding author upon request.

## Footnotes

1 When a series of related conditional discriminations are trained, equivalence relations emerge when the stimuli involved in those discriminations often become related to each other in ways that were not explicitly trained (e.g., when A1–B1 and B1–C1 are trained, C1–A1 emerges without explicit training).

2 Horne and Lowe (1996) defined intraverbal naming as the establishment of intraverbal relations between names of stimuli acquired during visual–visual conditional discrimination training.

3 In making this argument, we are not claiming that simultaneous naming procedures cannot generate derived (bidirectional) relational responding. Rather, we are simply suggesting that a nonsimultaneous procedure provides a more robust test for naming that involves derived relational responding.
